# History of the methodology of disease gene identification

**DOI:** 10.1002/ajmg.a.62400

**Published:** 2021-06-23

**Authors:** Stylianos E. Antonarakis

**Affiliations:** ^1^ University of Geneva Medical School Geneva Switzerland; ^2^ Medigenome, Swiss Institute of Genomic Medicine Geneva Switzerland

**Keywords:** gene identification, genetic methods, genomic variants, Mendelian disorders

## Abstract

The past 45 years have witnessed a triumph in the discovery of genes and genetic variation that cause Mendelian disorders due to high impact variants. Important discoveries and organized projects have provided the necessary tools and infrastructure for the identification of gene defects leading to thousands of monogenic phenotypes. This endeavor can be divided in three phases in which different laboratory strategies were employed for the discovery of disease‐related genes: (i) the biochemical phase, (ii) the genetic linkage followed by positional cloning phase, and (iii) the sequence identification phase. However, much more work is needed to identify all the high impact genomic variation that substantially contributes to the phenotypic variation.

## 1 INTRODUCTION

As of the time of this writing (April 20, 2021), there are 4692 protein‐coding genes with allelic variants causing Mendelian disorders in Online Mendelian Inheritance in Man (OMIM). The first gene shown to be causative for a Mendelian disorder was the β‐globin gene (*HBB* in today's nomenclature), pathogenic variants of which cause β‐thalassemia, and sickle‐cell disease. The *HBB* gene cluster was cloned and sequenced in the late 1970s (Fritsch et al., 1979) and many pathogenic variants were found in the following years (Fritsch et al., 1979; Orkin et al., 1982) and https://www.omim.org/entry/141900?search=HBB&highlight=hbb). Since then, there has been a tremendously successful research activity in the search for genes from Mendelian disorders; this has been fueled by the development and the infrastructure provided by the sequence structure and function of the human genome and that of model organisms. The interaction and synergy between technology development, laboratory research, computational capabilities, and clinical expertise resulted in the current evolution of medical care and promises a whole transformation of medicine in terms of diagnostic and treatment possibilities.

**FIGURE 1:**
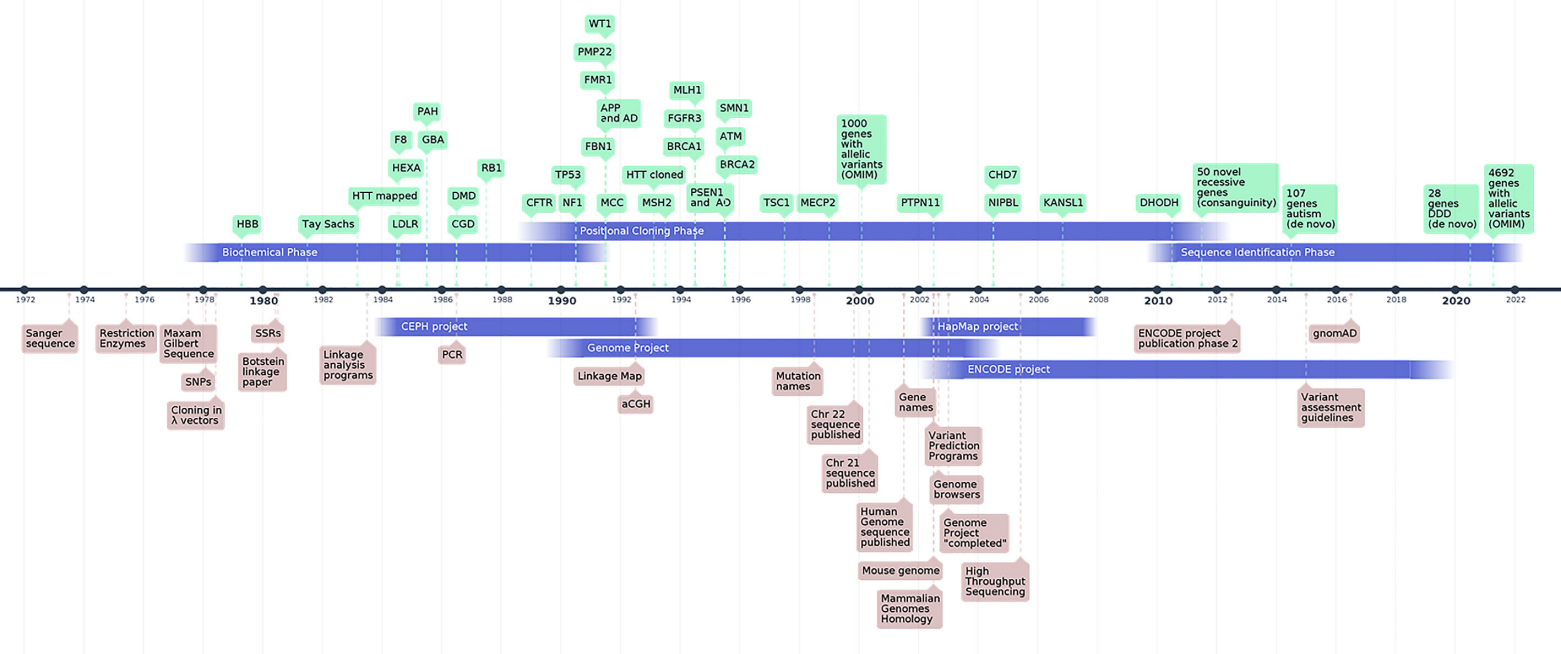
A timeline of events regarding gene identification for Mendelian disorders. The periods of projects are shown below, and the phases of gene discovery above the timeline, respectively. Below the timeline are also shown some selected events related to the gene identification process and methodology. Above the timeline are depicted some selected gene discoveries for Mendelian disorders

The pace of new gene‐disease link discoveries is presently roughly one per day. This could be seen on one hand as impressively fast compared to the 1980s when the discovery rate was one per 4–5 years, or on the other hand as depressively slow since at this rate we will probably need more than 25 years to find all protein‐coding gene links with (near)‐Mendelian disorders. In this short paper, I will discuss, with the bias of a Johns Hopkins prospective, some landmark events that have substantially influenced the discovery of the disease‐related genes. The timeline of Figure 1 provides a graphical representation of events discussed below.

## 2 PHASE 1: THE GLOBIN GENES DURING THE DAWN OF THE GENOME ERA; THE BIOCHEMICAL PHASE

An important place in the history of disease gene cloning and characterization is occupied by the beta and alpha globin genes responsible for the hemoglobinopathies including sickle cell disease and thalassemias. The beta and alpha globin genes (*HBB* (Fritsch et al., 1979) and *HBA* (Leder et al., 1978; Orkin, 1978)) were first cloned because of the abundance of their RNA transcript and encoded protein in red blood cells. Because blood is an easily accessible tissue, and sickle cell disease is a common disorder in various populations, much research attention has been directed toward understanding sickle cell disease. Advances in peptide sequencing resulted in identification of hemoglobin protein subunits and the pathogenic variant of the sickle hemoglobin many years before gene cloning (Ingram, 1959). In addition, the abundance of the alpha and beta globin RNAs in blood enabled the cloning of cDNA of the *HBA* and *HBB* genes in human and mouse. Methods for specific DNA cleavage by restriction endonucleases (see Nathans & Smith, 1975 for review and history), and gene cloning in lambda vectors (Maniatis et al., 1978), along with methods to determine the sequence of nucleic acids (Maxam & Gilbert, 1977; Sanger et al., 1973), provided the opportunity to identify the majority of beta and alpha thalassemia pathogenic variants. The study of these pathogenic variants provided a considerable background knowledge for the nature and consequences of mutations in human genes. Nonsense codons, missense codons, termination codon substitutions, splicing errors of various kinds (canonical dinucleotides of the donor and acceptor sites, cryptic site activation, novel splice sites), promoter regions, distal regulatory elements, microdeletions and microduplications, and mechanisms of unequal crossing over were some of the lessons from the study of mutations in the globin genes that served the subsequent identification of hundreds of disease genes (Antonarakis et al., 1985). In addition, DNA polymorphic variation around the beta‐globin gene provided the knowledge of haplotype structure, linkage disequilibrium, hot spots for recombination, and population‐specific mutation spectra (Antonarakis et al., 1982; Chakravarti et al., 1984). The haplotype structure of the beta‐globin gene cluster had a substantial impact in the choice of the candidate mutant alleles to be sequenced and the discovery of the full spectrum of pathogenic variants in a given population in the pre‐polymerase chain reaction (PCR) era (Orkin et al., 1982). In those days, the cloning of each gene in lambda vectors was labor‐intensive, and DNA sequencing was the privilege of a small number of research laboratories. Johns Hopkins was one of the major centers of the *HBB*‐related research, and many important discoveries in this field occurred there. Other examples of disease gene identification at the DNA level in the mid‐80's based on the protein sequence include among others the *LDLR* gene for familial hypercholesterolemia (Yamamoto et al., 1984), the *HEXA* gene for Tay‐Sachs disease (Myerowitz & Proia, 1984), the *GBA* gene for Gaucher disease (Sorge et al., 1985), the *F8* gene for Hemophilia A (Gitschier et al., 1984), and the *PAH* gene for phenylketonuria (Kwok et al., 1985).

## 3 GENOMIC VARIABILITY, GENOME INFRASTRUCTURE, LINKAGE ANALYSIS

The study of the *HBB* gene provided an initial appreciation of the considerable polymorphic variability of the human genome. Since YW Kan's discovery of the polymorphic *HpaI* restriction enzyme site 3′ to *HBB* (Kan & Dozy, 1978), thousands of such sites have been identified that were either biallelic (single nucleotide variants) or multiallelic (short sequence repeats (Wyman & White, 1980)) in the population. The extensive copy number variation was discovered later (see Freeman et al., 2006 for review). A seminal proposal by Botstein et al. (1980) published in 1980 provided the theoretical framework for a linkage of a disease‐related locus to a polymorphic marker in the genome, that is, that a disease‐related gene maps close to a polymorphic marker and therefore the chance for recombination between the two loci (the gene and the marker) in meiosis is minimal. In practice, that meant that given large families with sufficient number of affected individuals and the availability of a sufficient number of polymorphic markers, one could successfully map the unknown disease gene in a small interval of the human genome. This theoretical expectation was put to test in the real world: large pedigrees with the dominant Huntington disease (Gusella et al., 1983) were tested in a linkage analysis using a then small set of polymorphic markers detected by Southern blot, and the unknown locus for Huntington disease was mapped to chromosome 4! The success of this story fueled the efforts for mapping of elusive genes and subsequently cloning them by searching in the “neighborhood” of the linked marker. Computational linkage algorithms were introduced for wide use in 1983 (Ott, 1983); the first such program was published a few years earlier (Ott, 1976).

The infrastructure necessary to facilitate positional cloning (cloning by mapping) was the discovery of a large number of informative polymorphic markers, and the establishment of linkage maps for each chromosome in the early 1990s so that the disease‐related gene location could be narrowed down to roughly one megabase of DNA sequence (“A comprehensive genetic linkage map of the human genome. NIH/CEPH Collaborative Mapping Group,” 1992). The use of samples from the CEPH (Centre d'Etude du Polymorphism Humain (Dausset et al., 1990)) consortium initiated in 1984 was instrumental for the generation of these maps. Sufficiently dense linkage maps for each chromosome were produced (Donis‐Keller et al., 1987; Warren et al., 1989), while the HapMap project that began in the late 1990s provided a wealth of polymorphic markers and an appreciation of linkage disequilibrium blocks of the human genome (Gabriel et al., 2002). Linkage analyses were extensively used to place genes responsible for Mendelian phenotypes in a small genomic interval of approximately 1 Mb. In parallel, introduction of PCR technology (Saiki et al., 1986) in 1986 greatly facilitated the study of DNA sequences without requiring labor‐intensive cloning in various vectors. Finally, advances of the Human Genome Project (Lander et al., 2001; Venter et al., 2001), particularly in the delivery of the first draft of the Human Genome Sequence and that of genomes of model organisms such as the mouse (Mouse Genome Sequencing et al., 2002) further facilitated the positional cloning of genes responsible for Mendelian disorders, in many cases where there were no biochemical clues regarding the identity of the disease‐related gene.

## 4 PHASE 2: POSITIONAL CLONING

The positional cloning phase of disease gene identification was very fruitful, since the genes responsible for most common Mendelian disorders were cloned during this period. The success was based on knowledge of genome infrastructure (mostly the linkage map), that was developed, the methods for linkage analysis, the availability of a wealth of common DNA polymorphic sites, and the study of large families with a considerable number of affected individuals. In addition, the development of physical maps from libraries of cloned segments of the human genome, and chromosome and somatic cell data also facilitated the gene searches (Burke et al., 1987). The era of positional cloning lasted until the early 2000s (Botstein & Risch, 2003). On February 2, 2000, the OMIM database passed the 1000 mark on genes with allelic variants, that is, genes that when mutated cause Mendelian phenotypes (Antonarakis & McKusick, 2000). The first two disease genes cloned with positional cloning were the chronic granulomatous disease (Royer‐Pokora et al., 1986), and the X‐Linked Duchenne Muscular Dystrophy gene *DMD* (Koenig et al., 1987; Monaco et al., 1986). Additional success stories were the first cancer‐related Mendelian gene retinoblastoma (Fung et al., 1987) *RB1*, the *CFTR* gene for cystic fibrosis (Riordan et al., 1989; Rommens et al., 1989), the *TP53* gene in a cancer prone Li‐Fraumeni syndrome (Malkin et al., 1990), the Wilms tumor gene *WT1* (Pelletier et al., 1991), the *NF1* gene for neurofibromatosis 1 (Marchuk et al., 1991; Viskochil et al., 1990; Wallace et al., 1990), a colorectal polyposis gene (Kinzler et al., 1991), the *FBN1* gene for Marfan syndrome (Dietz et al., 1991), the *APP* gene, which was linked to one form of Alzheimer disease (Goate et al., 1991), the Fragile X gene (Verkerk et al., 1991) *FMR1*, the *PMP22* gene for one form of the Charcot–Marie–Tooth disease (Lupski et al., 1991), the *MECP2* gene for Rett syndrome (Amir et al., 1999), the *MSH2* and *MLH1* genes for hereditary colon cancer (Leach et al., 1993; Papadopoulos et al., 1994), the presenilin 1 gene *PSEN1* responsible for another familial form of Alzheimer disease (Sherrington et al., 1995), the breast and ovarian cancer genes (Miki et al., 1994; Wooster et al., 1995) *BRCA1* and *BRCA2*, the *ATM* gene for ataxia telangiectasia (Savitsky et al., 1995), the *FGFR3* gene for achondroplasia (Rousseau et al., 1994; Shiang et al., 1994), the *SMN1* gene for spinal muscular atrophy (Lefebvre et al., 1995), the *TSC1* gene for tuberous sclerosis (van Slegtenhorst et al., 1997), the *PTPN11* gene for one form of the Noonan syndrome (Tartaglia et al., 2001), the *NIPBL* gene for the Cornelia de Lange syndrome (Krantz et al., 2004; Tonkin et al., 2004), and the *CDH7* gene for the CHARGE syndrome (Vissers et al., 2004). The Huntington disease gene *HTT* (The Huntington's Disease Collaborative Research Group, 1993) was cloned in 1993, 10 years after the linkage of the gene to chromosome 4 in 1983 because this gene was located near the chromosome 4p terminus, which was composed of many repetitive elements where a considerable number of recombination events in meiosis occur. This has made the chromosome “walking” difficult. A special case of positional cloning was the identification of some causative genes that mapped in deleted regions of the genome identified by comparative genomic hybridization using arrays of oligonucleotide probes (aCGH). This method for the detection of copy number variation in the genome was introduced in 1992 (Kallioniemi et al., 1992), and is still used extensively in diagnostic laboratories. Two examples of gene identification through an aCGH abnormality include the identification of the *KANSL1* gene for the dominant Koolen‐De Vries syndrome (Zollino et al., 2012), and the *GRID2* gene causing one form of autosomal recessive Spinocerebellar Ataxia (Hills et al., 2013).

## 5 GENOME SEQUENCE, AND HIGH THROUGHPUT SEQUENCE

The next dramatic event in the effort to identify causative genes and variants for Mendelian disorders was completion of the sequence of the euchromatic fraction of the human genome (Lander et al., 2001; Venter et al., 2001). This milestone was the product of hundreds of people as part of an international collaborative and competitive effort and provided the infrastructure for navigation in the genome, which tremendously facilitated disease‐gene discovery. A cascade of events has followed the genome sequence: (i) development of methods for faster, cheaper, and more accurate sequence (massive parallel sequencing; https://www.illumina.com/science/technology/next-generation-sequencing.html); (ii) appreciation of the extensive variability of the sequences in individuals and populations; (iii) initial exploration of the likely functional elements (ENCODE project (ENCODE Project Consortium, 2012)); (iv) sequencing of the genomes of model organisms (Mouse Genome Sequencing et al., 2002), which boosted the functional characterization of regions of homology with the human genome (Dermitzakis et al., 2002) and thus the mapping of likely pathogenic variants in humans; (v) development of public databases of variants of a large number of individuals (Lek et al., 2016); and (vi) development of computational methods for assessment of potential pathogenicity (Ramensky et al., 2002) of mostly rare variants in different populations. Availability of genome browsers (Clamp et al., 2003; Karolchik et al., 2003), approval of gene names by the HUGO Gene Nomenclature Committee (Povey et al., 2001), and guidelines for the nomenclature of mutations (Antonarakis, 1998; den Dunnen & Antonarakis, 2000) greatly facilitated the communication among investigators and further enhanced the Mendelian gene discovery and description of pathogenic variants.

The evolving genomic infrastructure resulted in the development of strategies for the discovery of disease‐related protein‐coding genes. A renaissance of Mendelian genetics took place (Antonarakis & Beckmann, 2006), and this trend continues until today. Since 2003 more than 3500 additional disease‐related genes for Mendelian disorders have been identified, and the pace of the new discoveries continues with a rate of approximately 1 “novel” disease‐gene per day.

## 6 PHASE 3: SEQUENCE IDENTIFICATION

In the post‐genome era, sequencing methods and approaches dominate novel gene discovery. Genomic infrastructure has provided the opportunity to discover new Mendelian genes because of two phenomena that facilitate gene identification: de novo mutations and consanguinity.

De novo mutations are ones that occur in gametogenesis and are present in the new zygote. The observed mutation rate after sequencing genomes of parents and their offspring is on the order of 1 × 10^−8^ per gamete per generation (Kong et al., 2012). Most de novo variants occur during spermatogenesis in males, presumably because of replication errors (Kong et al., 2012; Rahbari et al., 2016; Sasani et al., 2019). Paternal age positively correlates with the number of de novo variants in the zygote. Roughly, 1.5 additional de novo mutations occur per year of paternal age. Thus, a man at 50 years of age gives 30 more “de novo” variants to the fetus than when he was 30 years of age. With the average mutation rate mentioned above, one expects approximately 60 new variants in each newborn, a number that increases with the father's age. Since the exome, that is, the protein‐coding fraction of the genome is approximately 1.5% of the total sequence, there is on average 1 de novo variant per exome in each new zygote. This additional mutation load per newborn contributes to the de novo occurrence of dominant disorders. Thus, trio sequence analysis, that is, sequencing of parental DNA and their offspring with a sporadic case of a suspected Mendelian disorder could identify candidate genes for dominant disorders by focusing on the de novo variants in the trio analysis. In fact, more than half of causative dominant variants in sporadic cases of Mendelian disorders (just one affected offspring per pedigree) are de novo variants (Deciphering Developmental Disorders, 2017). A large number of dominant Mendelian genes have been identified in the last 15 years, and this effort continues since the industrialized countries with even small numbers of children per family provide ample numbers of trios with sporadic occurrence of Mendelian phenotypes (Kaplanis et al., 2020). On April 20, 2021, OMIM contained 919 entries with the search term de novo in the text of allelic variants.

The other environmental phenomenon is the practice of consanguinity in a considerable fraction of some populations (Hamamy et al., 2011). Consanguinity, that is, unions of close relatives dramatically increases regions of homozygosity in an offspring's genome, and therefore brings together homozygous deleterious alleles that cause autosomal recessive disorders. It has been estimated that approximately 5% of the world's population practices consanguinity for religious, cultural, economic, traditional and other reasons (Hamamy et al., 2011). On average, a child of an outbred couple has 30 Mb of homozygosity in its genome; in contrast, a child of first‐cousin parents has approximately 10 times more regions of homozygosity in its genome, and thus there is an increased risk for a homozygosity of deleterious variants and higher incidence of recessive diseases (Antonarakis, 2019). Considerable efforts have thus been made in populations that practice consanguinity, and hundreds of novel recessive causative genes have been identified in this way (Monies et al., 2019). This effort continues since the majority of the unknown Mendelian genes are likely to be recessive. It has been estimated that approximately 7000 additional recessive genes have escaped detection (Antonarakis, 2019; Bamshad et al., 2019). This may be an underestimate since it is now recognized that a considerable number of protein‐coding genes if mutated could result in either dominant or recessive phenotypes, according to the functional consequences of the variants and the function of the encoded mutant protein. As of April 20, 2021, OMIM contained 1936 entries with the search term “*consanguineous*” in the text of allelic variants.

The first Mendelian gene identified using high‐throughput sequencing and appropriate filtering of the thousands of variants was the *DHODH* gene in Miller syndrome in 2010 (Ng et al., 2010). Since then thousands of novel gene‐disease links have been discovered mainly using exome but also genome sequencing; publications reporting discovery of tens of novel gene‐disease links were not uncommon (De Rubeis et al., 2014; Deciphering Developmental Disorders, 2017; Kaplanis et al., 2020; Najmabadi et al., 2011). As of April 20, 2021, OMIM contained 2015 entries with the search term “*exome*” in the text of allelic variants. Publication of guidelines for interpretation of variant pathogenicity (MacArthur et al., 2014; Richards et al., 2015) have also helped in the assessment of variants as causative of a Mendelian disease.

## 7 CHROMATIN DYSFUNCTION AND NONCODING REGIONS; THE NEXT FRONTIER?

The vast majority of the high impact variants that cause Mendelian disorders are in the coding regions and splice junctions of protein‐coding genes. However, some Mendelian disorders are due to pathogenic variants in distal regulatory elements or enhancers of genes. One such example is the discovery of pathogenic variants in an enhancer of the Sonic Hedgehog SHH gene that maps approximately 1 Mb away of the coding gene (Lettice et al., 2003). Furthermore, variants in genomic regions that modify chromatin interactions can also cause developmental abnormalities such as limb malformations (Franke et al., 2016; Lupianez et al., 2015). Variants in noncoding genes can also have a strong phenotypic impact as in the examples of a lncRNA in brachydactyly Type E (Maass et al., 2012), and the lncRNA gene Maenli in limb malformation (Allou et al., 2021). Therefore, whole genome sequence, RNA sequencing, chromatin interactions methods, epigenetic modification of DNA sequences, and histone modifications will contribute to the identification of additional genes and other functional genomic elements in Mendelian disorders. A full understanding of the molecular consequences of high impact variants is thus imperative for the introduction of rationalistic treatments based on the pathophysiology of each disease and the particularity of its molecular lesion. One illustrative example from our laboratory is the identification of the *SLC6A6* taurine transporter as the causative gene/protein for a recessive disease with progressive retinal degeneration and cardiomyopathy; this disease was successfully treated with oral taurine immediately after discovery of the causative gene (Ansar et al., 2020).

The work of thousands of investigators from different disciplines (laboratory, clinical, computational) over the last 50 years resulted in a triumph over the molecular understanding of Mendelian disorders. Participation of patients and their families has been crucial in the discovery of disease‐related gene variants. Use of animal and cellular models also contributed to these discoveries and to the understanding of the molecular pathophysiology of these disorders. Personalities such as Dr Victor McKusick were extremely influential during this period.

The future challenges are many: the exploration of the entire genomic variation of each individual, the function of each variant, the contribution of this variation to the phenotypic variation, and the therapy of rare and common genomic disorders both constitutional and somatic. As William Shakespeare said in the Tempest: “What's past is prologue.”

## Data Availability

Not applicable. There are no data in this manuscript.
